# Carbon dioxide stimulation of photosynthesis in *Liquidambar styraciflua* is not sustained during a 12-year field experiment

**DOI:** 10.1093/aobpla/plu074

**Published:** 2014-11-17

**Authors:** Jeffrey M. Warren, Anna M. Jensen, Belinda E. Medlyn, Richard J. Norby, David T. Tissue

**Affiliations:** 1 Climate Change Science Institute and Environmental Sciences Division, Oak Ridge National Laboratory, Oak Ridge, TN 37831-6301, USA; 2 School of Biological Sciences, Macquarie University, Sydney, NSW 2019, Australia; 3 Hawkesbury Institute for the Environment, University of Western Sydney, Richmond, NSW 2753, Australia

**Keywords:** Acclimation, down-regulation, free-air CO_2_ enrichment, nitrogen limitation, sweetgum.

## Abstract

Elevated atmospheric CO_2_ (eCO_2_) often increases photosynthetic CO_2_ assimilation (*A*) in field studies of temperate tree species. However, there is evidence that *A* may decline through time due to biochemical and morphological acclimation, and environmental constraints. Indeed, at the free-air CO_2_ enrichment (FACE) study in Oak Ridge, Tennessee, *A* was increased in 12-year-old sweetgum trees following 2 years of ∼40 % enhancement of CO_2_. *A* was re-assessed a decade later to determine if the initial enhancement of photosynthesis by eCO_2_ was sustained through time. Measurements were conducted at prevailing CO_2_ and temperature on detached, re-hydrated branches using a portable gas exchange system. Photosynthetic CO_2_ response curves (*A* versus the CO_2_ concentration in the intercellular air space (*C*_i_); or *A*–*C*_i_ curves) were contrasted with earlier measurements using leaf photosynthesis model equations. Relationships between light-saturated photosynthesis (*A*_sat_), maximum electron transport rate (*J*_max_), maximum Rubisco activity (*V*_cmax_), chlorophyll content and foliar nitrogen (N) were assessed. In 1999, *A*_sat_ for eCO_2_ treatments was 15.4 ± 0.8 μmol m^−2^ s^−1^, 22 % higher than aCO_2_ treatments (*P* < 0.01). By 2009, *A*_sat_ declined to <50 % of 1999 values, and there was no longer a significant effect of eCO_2_ (*A*_sat_ = 6.9 or 5.7 ± 0.7 μmol m^−2^ s^−1^ for eCO_2_ or aCO_2_, respectively). In 1999, there was no treatment effect on area-based foliar N; however, by 2008, N content in eCO_2_ foliage was 17 % less than that in aCO_2_ foliage. Photosynthetic N-use efficiency (*A*_sat_ : N) was greater in eCO_2_ in 1999 resulting in greater *A*_sat_ despite similar N content, but the enhanced efficiency in eCO_2_ trees was lost as foliar N declined to sub-optimal levels. There was no treatment difference in the declining linear relationships between *J*_max_ or *V*_cmax_ with declining N, or in the ratio of *J*_max_ : *V*_cmax_ through time. Results suggest that the initial enhancement of photosynthesis to elevated CO_2_ will not be sustained through time if N becomes limited.

## Abstract (precis)

Atmospheric carbon dioxide levels have increased by∼25% over the last 50 years. While more carbon dioxide can initially stimulate plant photosynthesis, we found that long-term (12 years) exposure of sweetgum trees to elevated carbon dioxide resulted in no stimulation of photosynthesis. The loss of initial increases in photosynthesis was due to low leaf nitrogen levels, which suggests other limiting resources may moderate future impacts of elevated carbon dioxide on photosynthesis.

## Introduction

In 2013, annual atmospheric CO_2_ concentration exceeded 396 ppm at Mauna Loa, 2.6 ppm greater than in 2012 and 25 % greater than the initial measurements in 1959 (Keeling *et al.* 2014). Global atmospheric model simulations estimate that this trajectory will continue (IPCC 2013), and thus terrestrial plant communities will remain exposed to increasing CO_2_ for the foreseeable future. Atmospheric CO_2_ is a potent climate-forcing agent contributing to increased atmospheric temperatures, and ecosystem balance between plant CO_2_ uptake through photosynthesis and release through respiratory activity remains a key uncertainty in mechanistic Earth system models that project ecosystem feedbacks to the atmosphere. To address ecosystem response to elevated CO_2_ (eCO_2_), various plant species have been exposed to air enriched with CO_2_ in short-term open- or closed-topped field chambers. Results suggested substantial eCO_2_ stimulation of photosynthesis (Luxmoore *et al.* 1993; Gunderson and Wullschleger 1994; Medlyn *et al.* 1999; Norby *et al.* 1999) across various woody species (e.g. in oak, yellow poplar (Gunderson *et al.* 1993), pine (Tissue *et al.* 1997), eucalypts (Ghannoum *et al.* 2010) and tropical seedlings (Ziska *et al.* 1991)). In other studies, eCO_2_ induced significant down-regulation and loss of photosynthetic capacity (e.g. in Arctic tundra grasses and shrubs (Tissue and Oechel 1987; Oechel *et al.* 1994), beech (Epron *et al.* 1996) and spruce (Marek *et al.* 1995)), indicating that interaction with soil resources, longer term feedbacks and progressive plant acclimation remained a key uncertainty (Curtis and Wang 1998). New, larger experiments using free-air CO_2_ enrichment (FACE) technology (Norby *et al.* 2001) have allowed field crops, grasses and, in particular, woody forest vegetation to be exposed to eCO_2_ over many years. Such studies allow distinction of long-term responses from transient responses due to leaf plasticity, stand development or inter-annual environmental variability.

Similar to the response of plants in chambers, an initial increase in net photosynthesis (*A*) and net primary productivity is common across woody plant species exposed to FACE eCO_2_ treatments (Norby *et al.* 2005; Ainsworth and Rogers 2007), including in *Liquidambar styraciflua* (Sholtis *et al.* 2004), *Pinus taeda* (Crous and Ellsworth 2004), *Populus × euramericana*, *Populus alba*, *P. nigra* (Bernacchi *et al.* 2003; Liberloo *et al.* 2007), *P. tremuloides* (Noormets *et al.* 2010), *Fagus sylvatica*, *Quercus petraea*, *Carpinus betulus*, *Acer campestre* and *Tilia platyphyllos* (Bader *et al.* 2010). However, longer term eCO_2_ field results are mixed, with some experiments reporting feedbacks such as acclimation and photosynthetic down-regulation through time (Rey and Jarvis 1998; Medlyn *et al.* 1999; Kubiske *et al.* 2002; Bernacchi *et al.* 2003; Crous *et al.* 2008) and others not (Bader *et al.* 2010; Darbah *et al.* 2010).

Leaves typically adjust their Rubisco activity or content to the prevailing CO_2_ concentration, i.e. down-regulation by elevated CO_2_ (Tissue *et al.* 1993; Drake *et al.* 1997; Stitt and Krapp 1999; Rogers and Ellsworth 2002). Since eCO_2_ increases the efficiency of Rubisco, *A* can be maintained or enhanced despite reductions in enzyme content, activity or maximum photosynthetic capacity. This implies greater photosynthetic N-use efficiency (PNUE) as displayed across C3 woody plant species exposed to elevated CO_2_ (Peterson *et al.* 1999; Calfapietra *et al.* 2007).

Sweetgum (*L. styraciflua*) is a common temperate North American tree species, and is the dominant canopy species at the ORNL-FACE site in TN, USA, and the primary mid-canopy species at the Duke-FACE site in NC, USA. Prior results from these sites indicate a strong and consistent enhancement of light-saturated photosynthesis (*A*_sat_) by elevated CO_2_; the ∼40 % increase in atmospheric CO_2_ resulted in 45 % enhancement of *A*_sat_ at the ORNL-FACE site after 3 years of treatments (Gunderson *et al.* 2002; Sholtis *et al.* 2004) and consistently >50 % enhancement of *A*_sat_ at the Duke-FACE site through 6 years of treatments (Herrick and Thomas 1999, 2003; Springer *et al.* 2005). After 3 years of treatment at ORNL-FACE there was no change in maximum photosynthetic capacity (*A*_max_), maximum electron transport rate (*J*_max_) or maximum carboxylation rate (*V*_cmax_) when foliage from both treatments was measured at the same CO_2_ concentrations (Sholtis *et al.* 2004). Elevated CO_2_ did reduce foliar N content and increase soluble carbohydrates and leaf mass per area (LMA, Herrick and Thomas 2003; Sholtis *et al.* 2004)—results consistent across time for sweetgum in both FACE studies. After 6 years of eCO_2_ treatment at ORNL-FACE, there was still no significant difference in *A*_sat_ when both treatments were measured at 400 ppm (Monson *et al.* 2007), indicating little eCO_2_ down-regulation of photosynthesis.

After 12 years of eCO_2_ treatment at the ORNL-FACE research study, we re-assessed photosynthetic capacity of the dominant sweetgum trees to determine if the early enhancement of photosynthesis during the years 1–3 (Gunderson *et al.* 2002; Sholtis *et al.* 2004) was sustained after an additional 9–10 years of treatment.

Over the 12-year study, the canopy spread upward and there was expansion of the stand diameter distribution as dominant individual trees maintained growth, while suppressed individuals stagnated and began to die. In addition, soil N availability decreased across the site, with the greatest rates of decline in the eCO_2_ plots (Garten *et al.* 2011). Decreased site nutrient availability was reflected by persistent inter-annual reductions in foliar N content and net primary production (NPP) (Norby *et al.* 2010), and a shift in carbon (C) allocation belowground to roots (Norby *et al.* 2004). The reductions in NPP through time were greatest for eCO_2_ plots despite treatment-specific increases or decreases in canopy leaf area in response to inter-annual variability such as drought (Warren *et al.* 2011*b*), or the substantial reduction in eCO_2_ site water use (Warren *et al.* 2011*a*, *b*). In addition to increased soil moisture availability in eCO_2_ plots, the increase in root production should lead to an enhanced capacity for soil nutrient extraction. Yet canopy N content continued to decline through time at a greater rate for eCO_2_ plots than for aCO_2_ plots, which correlated with a greater reduction in NPP for eCO_2_ plots (Norby *et al.* 2010).

The main goal of this project was to determine if the initial enhancement of photosynthesis by eCO_2_ was sustained through time, and if responses were linked to the progressive decline in site resource availability (Garten *et al.* 2011), foliar N content (Norby *et al.* 2010) and shift in internal plant C allocation (Norby *et al.* 2004). In 2008–09, we re-measured gas exchange in sweetgum leaves and compared the results with data from previous gas exchange campaigns conducted across seasons from 1998 to 2000 (Gunderson *et al.* 2002; Sholtis *et al.* 2004); specifically the mid-summer 1999 dataset was used as it was the most comprehensive dataset and overlapped with the same period sampled in 2008–09 (from late July to early August). We hypothesized that (i) declining N availability would be reflected in reduced photosynthetic capacity through time and (ii) when photosynthesis is N-limited, eCO_2_ leaves maintain greater RuBP-regeneration capacity, at lower Rubisco carboxylation rates, compared with aCO_2_ leaves (e.g. the slope of *J*_max_ : *V*_cmax_ is steeper in aCO_2_ leaves). Finally, we evaluated treatment effects on coupling between stomatal conductance and C-assimilation.

## Methods

### Site description and CO_2_ treatments

The study site was a 20-year-old sweetgum (*L. styraciflua* L.) plantation forest in Oak Ridge National Environmental Research Park in eastern TN, USA (35°54′N; 84°20′W). The soil was an Aquic Hapludult with a silty clay-loam texture. A free-air CO_2_ enrichment system (Hendrey *et al.* 1999) was installed at the site in four 25-m-diameter plots in 1996. The FACE system regulated the release of CO_2_ from vertical PVC pipes located in a ring around each plot based on wind speed, wind direction and in-situ measurements of current CO_2_ concentration within the canopy. From 1998 to 2009, CO_2_ was released into two FACE rings during each growing season to a target [CO_2_] of 560 ppm. Two FACE rings received ambient [CO_2_] and one additional ring, without FACE infrastructure, was established to serve as a third control plot. Actual CO_2_ enrichment varied through time as ambient concentrations rose incrementally and through modifications in the release regime, which resulted in a mean 40 % increase over ambient CO_2_. In 2008, tree height ranged from 10 to 24 m (mean = 18.2 ± 3.4 m (±1 SD)), median tree diameter was 14.6 ± 4.1 cm (±1 SD) and peak leaf area index (mid-July) was 4.1 ± 0.2. Mean growing season (April–October) temperature was 19.6 °C in 2008 and 19.1 °C in 2009, and growing season precipitation was 440 mm in 2008 and 511 mm in 2009 (Riggs *et al.* 2010). The site, experimental design and FACE apparatus have been previously described (Norby *et al.* 2001; Warren *et al.* 2011*b*) and results including micrometeorological data have been archived for public use: http://cdiac.ornl.gov/ftp/FACE/ornldata.

### Photosynthesis

For each measurement campaign (2008–09), branches were collected early in the morning from mid-canopy (2009) and fully exposed upper canopy (2008, 2009) dominant or co-dominant trees within each treatment plot. Canopy access was achieved via the FACE infrastructure towers, which provided access to trees within ∼8 m from the towers. Trees were selected primarily from within the treatment plots (i.e. >2.5 m from the ring edge); however, in ambient plots, several buffer trees were also sampled. On measurement days, 1–2 m long branches were cut from the mid- and upper canopy of selected trees with a pole pruner and quickly placed into plastic bags with wet paper towels to minimize desiccation. Branches were re-cut under water to remove potential embolism induced during removal from the trees. Most *L. styraciflua* vessel length is <0.3 m, therefore >0.5 m of the branch was removed. Prior measurements of gas exchange on severed or attached branches at the site found no differences in measurements over a 2-h period (Tissue *et al.* 2002; Monson *et al.* 2007). Gas exchange was measured using four portable photosynthesis systems (LI-6400XT, LI-COR, Lincoln, NE, USA) during late July 2008 and early August 2009. Measurements were conducted outside under ambient conditions on one or two leaves per branch (total samples *n* = 16–23 (2008); *n* = 29–31 (2009)). Conditions in the gas exchange cuvette were set to approximate ambient outside temperature: relative humidity was ∼50–80 %, photosynthetically active radiation was 1800 µmol m^−2^ s^−1^ and CO_2_ concentration was initially set at 400 ppm prior to the *A–C*_i_ curve measurements (see below). Foliage was retained for the analysis of N, chlorophyll and LMA. Similar raw datasets were available from previous work performed at the site in late July–August 1999 (Gunderson *et al.* 2002; Sholtis *et al.* 2004). At that time, photosynthesis was measured in situ using vertical man lifts. Upper canopy photosynthetic and biochemical datasets were combined across years (1999, 2008, 2009) to investigate shifts in response through time.

In addition to measurement of the light-saturated photosynthetic rate (*A*_sat_) at the CO_2_ concentration in which the trees were growing, response curves of assimilation versus the CO_2_ concentration within the intercellular spaces of the leaf (*C*_i_), or *A–C*_i_ curves, were measured in all years. These response curves were used to estimate maximum electron transport rate, *J*_max_, and maximum Rubisco activity, *V*_cmax_, using a consistent set of leaf photosynthesis model equations (Medlyn *et al.* 2002). In 2008 and 2009, curves were conducted through an initially declining, then increasing, reference CO_2_ regime (400, 300, 200, 100, 50, 400, 550, 700, 900, 1200, 1600 ppm). This contrasts slightly with curves conducted in 1999 using a declining CO_2_ regime (1500, 1200, 960, 760, 560, 360, 250, 175, 100, 50, 0 ppm). To contrast gas exchange responses at growth CO_2_, results were assessed at 400 or 550 ppm for aCO_2_ or eCO_2_, respectively. Since those CO_2_ concentrations were not part of the 1999 campaigns, values at 400 or 550 ppm were interpolated from the linear portion of each response curve (*R*^2^ > 0.99) for comparison. Atmospheric CO_2_ varied between 1998 and 2008 due to incremental ambient CO_2_ increases and FACE performance and management; range (384–405 or 528–560 ppm CO_2_) for aCO_2_ or eCO_2_, respectively. Relationships between *J*_max_, *V*_cmax_, stomatal conductance (*g*_s_), foliar N, chlorophyll content and CO_2_ treatments were assessed.

### Foliar biochemistry and LMA

To assess foliar chlorophyll and N concentrations, and LMA, nine leaf discs (9 mm diameter) were collected from the same area of each leaf (avoiding midrib) used for gas exchange measurements (2008–09). For chlorophyll analysis, two leaf discs were immediately placed in scintillation vials containing 5 mL of *N*,*N*-dimethylformamide (DMF) and extracted at 4 °C in the dark until analysis. Following extraction total chlorophyll, chlorophyll *a* and chlorophyll *b* were determined based on spectroscopy at 647 and 665 nm (Inskeep and Bloom 1985), and results contrasted with previous chlorophyll analysis in 1999 using an ethanol extraction (Sholtis *et al.* 2004). The two extraction solvents have been shown to provide comparable estimates of total chlorophyll in 9 of 11 tree species studied (Minocha *et al.* 2009). However, paired comparisons at our lab with sweetgum found that while DMF was able to fully extract chlorophyll within 1 day, 95 % ethanol at room temperature took up to 7 days for full extraction, and even then yielded only 80 % of the total chlorophyll yielded by DMF. Chlorophyll was thus likely underestimated in the earlier studies, and results are discussed in this context. Foliar N concentration was assessed for oven-dry (70 °C) discs using an elemental analyser (Costech Analytical Technologies, Inc., Valencia, CA, USA). In 1999, N content was measured on whole leaves, but not leaf discs, as used in 2008–09. Thus, comparison of N content across years required an LMA adjustment from whole leaf to leaf disc (LMA_disk_ = 0.9635(LMA_total_) + 0.7013 (mg cm^−2^); *n* = 28; *R*^2^ = 0.66; based on 2008 foliage). All N content is provided on a disc basis.

### Statistics

Regression slopes and treatment differences in photosynthesis, chlorophyll content, *V*_cmax_ and *J*_max_ were analysed using *t*-tests and analysis of variance techniques. There were no significant plot (ring) effects on chlorophyll, N, *J*_max_ or *V*_cmax_ measured in 1999 or later years (0.1 < *P* < 0.9). As such, individual leaves were considered as the (pseudo-replicated) experimental unit (df = 37–60 per treatment per year) as opposed to use of treatment rings (df = 1–2). This is common for analyses of these large, expensive studies where true replication is limited, and results should be considered in this context; i.e. there is a greater chance to detect *spurious* treatment effects (Type I errors) (Hurlbert 1984). Data manipulation and statistical procedures were completed using SAS statistical software (ver. 9.1.3, SAS Institute, Cary, NC, USA) and SigmaPlot (ver. 11.1, Systat Software, Inc., San Jose, CA). Relative differences between treatments and significance levels (*P* values) are presented; *P* < 0.05 indicates statistical significance.

## Results

**Figure 1.**
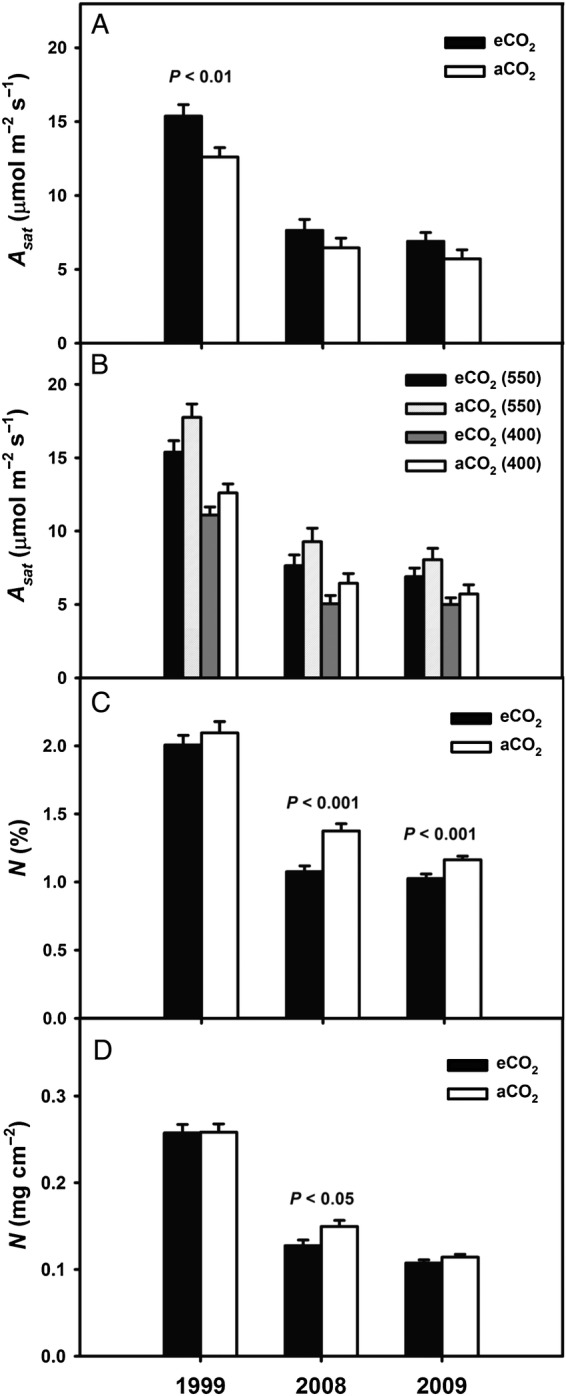
(A) Light-saturated photosynthesis (*A*_sat_) at growth CO_2_ (400 or 550 ppm for ambient (aCO_2_) and elevated (eCO_2_) CO_2_ treatments, respectively) for mature upper canopy sweetgum foliage in mid-summer 1999, 2008 and 2009 as derived from *A–C*_i_ curves. (B) *A*_sat_ for aCO_2_ and eCO_2_ foliage at 400 and 550 ppm CO_2_. (C) Foliar N content expressed on a mass (%) or (D) leaf area (mg cm^−2^) basis through time.

There were significant changes in photosynthetic rates and the photosynthetic response to eCO_2_ over time. In 1999, light-saturated photosynthesis (*A*_sat_) was significantly greater in eCO_2_ than in aCO_2_ foliage, but when re-measured in 2008, *A*_sat_ was similar for the two treatments. In 1999, *A*_sat_ was 15.4 µmol m^−2^ s^−1^ for eCO_2_ foliage, 22 % greater than for aCO_2_ foliage (*P* < 0.01; df = 60) (Fig. 1A). In both CO_2_ treatments, *A*_sat_ declined through time to 50 % of the 1999 rates by 2008 and to 45 % of the 1999 rates by 2009. In 2008 and 2009, there was no longer a significant difference in *A*_sat_ (*P* = 0.27, df = 37 (2008); *P* = 0.17; df = 56 (2009)) (Fig. 1A). There was no significant difference between treatments when *A*_sat_ was measured at the same CO_2_ concentrations, although measured *A*_sat_ tended to be lower for eCO_2_ foliage than for aCO_2_ foliage. In 1999, measured *A*_sat_ was 13 % lower for eCO_2_ foliage than for aCO_2_ foliage when both were measured at 550 ppm CO_2_ (*P* = 0.07; df = 60) and 12 % lower when both were measured at 400 ppm CO_2_ (*P* = 0.09) (Fig. 1B). In 2008, measured *A*_sat_ was 27 % lower for eCO_2_ foliage than for aCO_2_ foliage when both were measured at 550 ppm CO_2_ (*P* = 0.17; df = 37) and 22 % lower when both were measured at 400 ppm CO_2_ (*P* = 0.12). In 2009, measured *A*_sat_ was 24 % lower for eCO_2_ foliage than for aCO_2_ foliage when both were measured at 550 ppm CO_2_ (*P* = 0.24; df = 56) and 23 % lower when both were measured at 400 ppm CO_2_ (*P* = 0.35) (Fig. 1B).

Tracking the temporal pattern of *A*_sat_, foliar N concentration declined by ∼50 % from 1999 to 2009 across both treatments, with values as low as 6.9 or 8.5 mg g^−1^ for eCO_2_ or aCO_2_, respectively, which was not much higher than the seasonal senescent foliar litter (5.8 mg g^−1^). Mass-based foliar N content in eCO_2_ leaves was significantly lower than for aCO_2_ in 2008 and 2009 (*P* < 0.001), but not in 1999 (*P* = 0.22) (Fig. 1C). Elevated CO_2_ area-based N content was significantly lower than for aCO_2_ in 2008 (*P* < 0.05), but not in 2009 (*P* = 0.08) or in 1999 (*P* = 0.47) (Fig. 1D).

**Figure 2.**
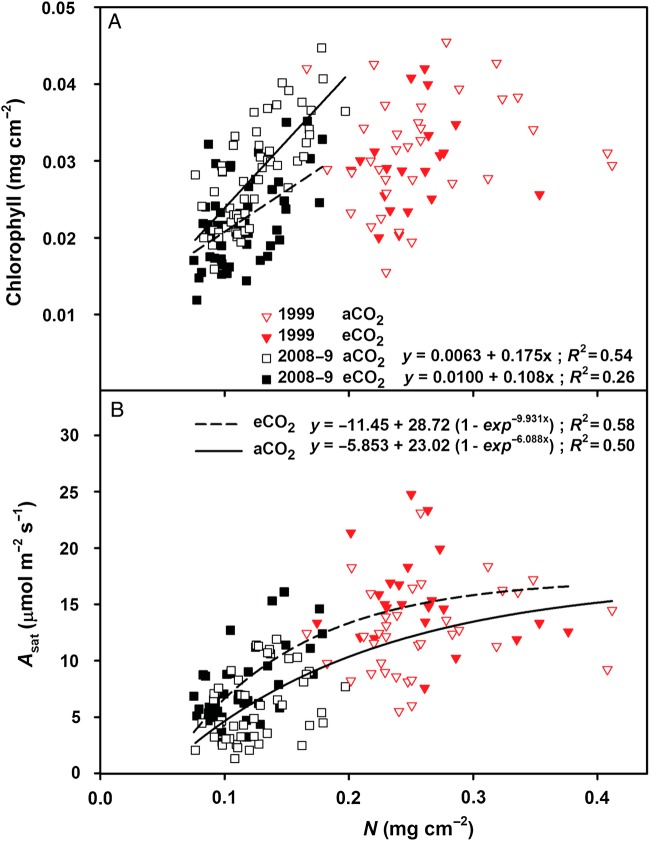
(A) Total foliar chlorophyll (chlorophyll *a* + *b*) for ambient (aCO_2_—solid line) and elevated (eCO_2_—dashed line) treatments in mid-summer 1999, 2008 and 2009 in relation to foliar N content, expressed on a leaf-area basis. Regressions were significant for *N* < 0.2 mg cm^−2^ (*R*^2^ = 0.26 or 0.54; *P* < 0.0001), and there was a significant treatment effect on slope (*P* < 0.05; *F* = 4.09; df = 107), but little trend was apparent in 1999 when *N* > 0.2 (*R*^2^ = 0.02 or 0.08; *P* > 0.05). (B) Light-saturated photosynthesis at growth CO_2_ (400 or 550 ppm) in mid-summer across years (1999, 2008, 2009) in relation to foliar N content, expressed on an area basis.

Many of the measured photosynthetic parameters were strongly related to N content, which dropped significantly over the course of the experiment. In 1999, the N content of foliage from both the treatments ranged from ∼0.2 to 0.4 mg cm^−2^, and there was no relationship between foliar N and chlorophyll content (Fig. 2A). When N content dropped <0.2 mg cm^−2^ (2008–09), there was a strong linear relationship with chlorophyll content, with a statistically steeper regression slope for the aCO_2_ foliage (Fig. 2A). Mean chlorophyll values were not different between CO_2_ treatments in 1999 (0.031 mg cm^−2^; *P* = 0.85), but were significantly lower for eCO_2_ (0.022 mg cm^−2^) than for aCO_2_ (0.028 mg cm^−2^; *P* < 0.001) foliage in 2008–09. Leaf mass per area also declined through time for both treatments, and was always greater for eCO_2_ foliage. In 1999, LMA was 8.6 % greater for eCO_2_ foliage (128.9 g m^−2^) than for aCO_2_ foliage (118.7 g m^−2^; *P* < 0.01). In 2008, LMA was not significantly greater for eCO_2_ foliage (115.9 g m^−2^) than for aCO_2_ foliage (107.7 g m^−2^; *P* = 0.10). In 2009, LMA was 6.6 % greater for eCO_2_ foliage (104.5 g m^−2^) than for aCO_2_ foliage (98.0 g m^−2^; *P* < 0.01).

Nitrogen content also had a significant impact on *A*_sat_, particularly at lower values of N. Values of *A*_sat_ plateaued as N content increased (Fig. 2B). The linear regression slope between *A*_sat_ and N for 1999–2009 was slightly steeper for eCO_2_ than for aCO_2_ (*P* = 0.055) slopes and coincided at 0.057 mg cm^−2^, which is approximately the N content of senescent tissue (regression not shown).

**Figure 3.**
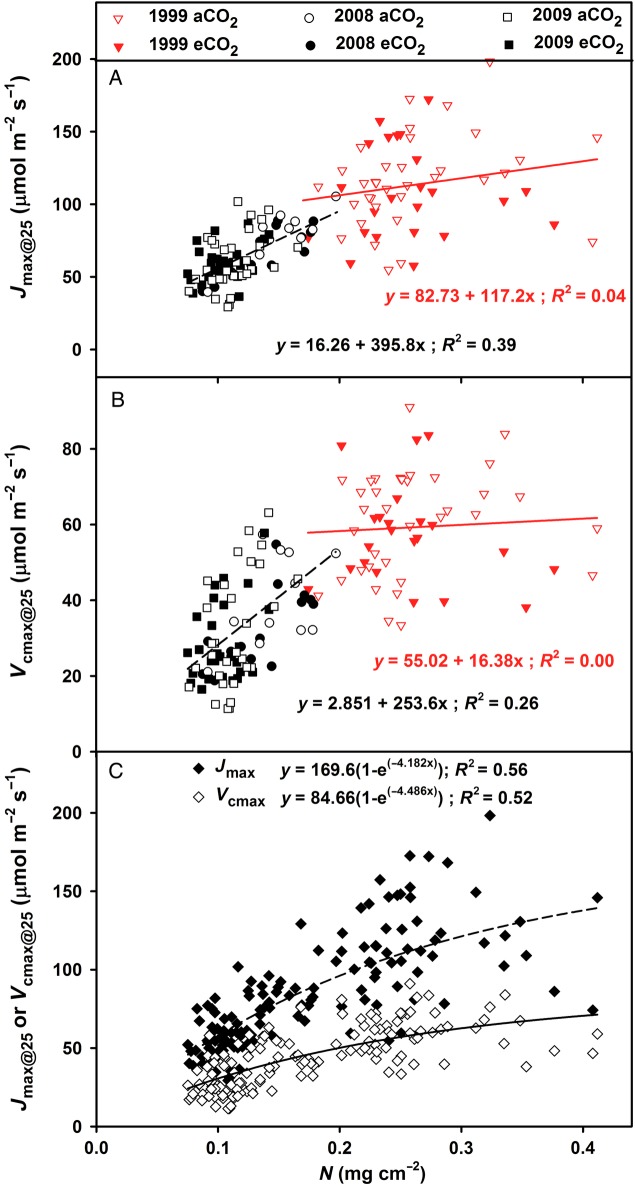
(A) Maximum electron transport rate (*J*_max_) and (B) carboxylation rate (*V*_cmax_) at 25 °C for ambient (aCO_2_) and elevated (eCO_2_) CO_2_ treatments by year or (C) across all years in relation to foliar N content on an area basis (as leaf discs). Linear regressions by treatment were generally significant in 2008 and 2009 (*R*^2^ = 0.14–0.80; *P* = 0.07–0.003) when *N* < 0.2 mg cm^−2^, but not significant in 1999 (*R*^2^ = 0–0.08; *P* = 0.98–0.1). There were no significant treatment effects on the relationships within or across years (*P* = 0.22–0.75). Regression equations are given for combined data across treatments.

**Figure 4.**
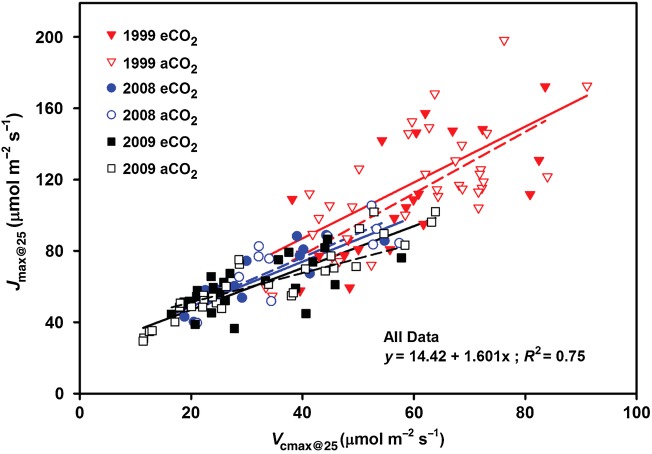
Relationships between maximum carboxylation rate (*V*_cmax_) and electron transport rate (*J*_max_) standardized to 25 °C for ambient (aCO_2_—solid lines) and elevated (eCO_2_—dashed lines) CO_2_ treatments through time. Regressions were significant for all treatments and years (*R*^2^ = 0.45–0.77), but there were no treatment effects on the relationships (in 2009, aCO_2_ had a slightly steeper slope, *P* = 0.067).

Values of *J*_max_ and *V*_cmax_ were not related to N content above ∼0.2 mg cm^−2^, but declined strongly with declining N content (Fig. 3A and B). There was a minimal overlap in N content of leaf samples from early (0.166–0.412 mg cm^−2^) to late (0.075–0.197 mg cm^−2^) years, which introduces a potential confounding effect of N × year, and which prohibited further refinement of the ∼0.2 mg cm^−2^ N response threshold. Even so, annual measurements throughout the study confirm the N differences as foliage displayed an incremental decline in N content through time (Norby *et al.* 2010). There were no CO_2_ treatment differences in the relationship between N content and *J*_max_ or N content and *V*_cmax_ <0.2 mg cm^−2^. Exponential rise-to-max regressions through all data provided a good fit for *J*_max_ or *V*_cmax_ with N content, and again suggested N saturation for the 1999 foliage (Fig. 3C). While there was no CO_2_ treatment effect on the ratio of *J*_max_ : *V*_cmax_ through time (Fig. 4), the slope of the linear regression of *J*_max_ : *V*_cmax_ declined through time across treatments (*P* < 0.05) from ∼1.6 in 1999 to ∼1.0 in 2009, indicating a decline in electron transport rate with respect to carboxylation rate through time. There was no relationship between *J*_max_ : *V*_cmax_ and N content (*R*^2^ = 0.02).

**Figure 5.**
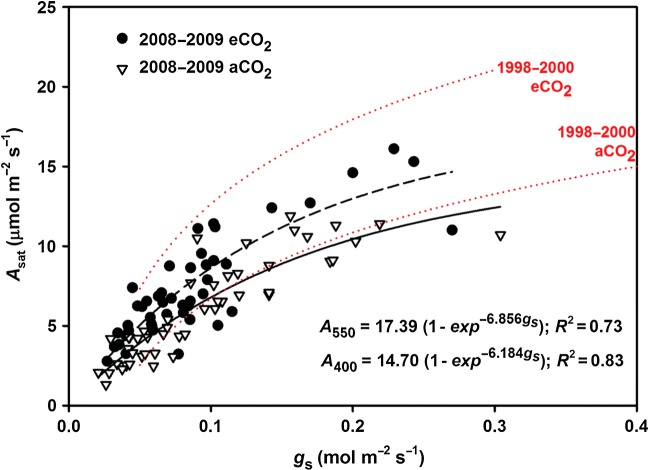
Light-saturated photosynthesis (*A*) in relation to stomatal conductance (*g*_s_) for mature upper canopy foliage exposed to elevated (eCO_2_; dashed line) or ambient (aCO_2_; solid line) CO_2_ (400 or 550 ppm, respectively) in mid-summer 2008 and 2009. Dotted regression lines represent results of similar measurements conducted across seasons from 1998 to 2000 based on Gunderson *et al.* (2002).

Photosynthetic water-use efficiency (WUE, photosynthesis per unit water use; *A*_sat_ : *g*_s_) in 2008–09 was ∼35 % greater for eCO_2_ foliage than for aCO_2_ foliage (*P* = 0.02; Fig. 5). The relationship between *A*_sat_ and stomatal conductance (*g*_s_) was similar to aCO_2_ foliage measured earlier (1998–2000) during different seasons and across years, but not for eCO_2_ foliage measured earlier (Fig. 5—regression lines).

## Discussion

Photosynthesis was down-regulated after long-term eCO_2_ treatments at the ORNL-FACE research site due to a reduction in foliar N content, a symptom indicative of progressive N limitation as the stand developed (Norby *et al.* 2010). Net photosynthesis (*A*) and photosynthetic capacity of sweetgum trees declined through time as the stand matured; these responses were correlated with increased nutrient (N, Ca^2+^, Mg^2+^) sequestration in long-term biomass or soil organic matter and reduced availability and uptake of N (Finzi *et al.* 2006; Norby *et al.* 2010; Iversen *et al.* 2011; K. Kim *et al.*, unpubl. data). Consistent with our first hypothesis, we found a significant reduction in overall photosynthetic capacity across treatments as leaf N content declined. However, in contrast to our second hypothesis, the reduction in foliar N was so acute that the benefit of eCO_2_ in generating enhanced photosynthetic efficiency was lost. As such, the initial CO_2_ stimulation (∼45 % greater) of net photosynthesis observed after 3 years (Gunderson *et al.* 2002; Sholtis *et al.* 2004) was not sustained after 12 years.

There was no relationship between foliar N and *A* in 1999, suggesting that foliar N content of upper canopy leaves (2.0–2.1 %) was in excess of photosynthetic requirements during the early years of the experiment. Total canopy N content in aCO_2_ plots declined from ∼1.8 % prior to the experiment in 1996 to 1.6 % by 2004 (Norby *et al.* 2010). Earlier work in younger 6-year-old sweetgum stands indicated that a leaf N threshold for maximum biomass production was ∼1.9 % (Scott *et al.* 2004). In March 2004, part of our sweetgum stand was fertilized (200 kg ha^−1^ urea), which boosted upper canopy leaf N concentrations from ∼1.65 % (control) to 2.04 % (fertilized) by late July, and substantially increased canopy leaf area and stem growth (Iversen and Norby 2008). The sweetgum trees at the Duke-FACE site indicated no down-regulation of photosynthesis and no change in tissue N content of upper canopy leaves from 1997 to 2004 (N content >1.5 %) (Herrick and Thomas 1999; Tissue *et al.* 2002; Springer *et al.* 2005; Ellsworth *et al.* 2012). Together, these prior studies support our conclusion that upper canopy photosynthesis at ORNL-FACE was not N limited in 1999, but strongly limited by the end of the experiment when the foliar N content had dropped to 1.03 % (eCO_2_) and 1.16 % (aCO_2_), which was significantly lower for the eCO_2_ treatment.

There was also no relationship between foliar N and chlorophyll in 1999, suggesting that the chlorophyll content was in excess of photosynthetic requirements during the early years of the experiment. However, the chlorophyll values reported for the site in 1999 appear to be too low, suggesting a poor extraction yield occurred with the ethanol solvent as described earlier in the Methods section: ‘Foliar biochemistry and leaf mass per area’. Chlorophyll values ranged from ∼0.020 to 0.043 mg cm^−2^ in 1999, similar to the values in 2008–09 (0.014–0.041 mg cm^−2^), despite much lower N content. Actual chlorophyll levels at the ORNL-FACE site in 1999 were thus likely significantly higher than indicated here. Indeed, chlorophyll extracted from similar aged sweetgum trees at the Duke-FACE site that were not N limited indicated late season values from 0.047 to 0.071 mg cm^−2^ (Herrick and Thomas 2001).

The lack of relationship between foliar N content and chlorophyll, *J*_max_ or *V*_cmax_ in 1999 suggests that the photosynthetic apparatus in sweetgum was N saturated above ∼0.2 mg cm^−2^ and that N had accumulated in excess to meet other N requirements (e.g. growth and storage). A similar pattern of N saturation of *V*_cmax_ has been suggested for pine at the Duke-FACE site, with saturation of the carboxylation rate at tissue contents >1.4 % N (Palmroth *et al.* 2013). Nitrogen is most often stored as free amino acids or proteins, especially Rubisco, or as inorganic N within the vacuole (Proe and Millard 1994; Warren and Adams 2004). It is likely that reductions in enzyme activation state in eCO_2_ foliage can partially uncouple photosynthetic response from foliar N content, as N is shifted into non-active ‘storage’ components. For example, the activation state of Rubisco has been shown to decline linearly with increasing leaf N in apple (Cheng and Fuchigami 2000). Given that *J*_max_ and *V*_cmax_ are often modelled based on N content of the leaf, not on the proportion of N actively used in photosynthesis this may lead to increased uncertainty in estimates of GPP at higher N contents.

While the initial foliar N content of the stand was adequate for photosynthesis, N became progressively more limiting, restricting light harvesting, rates of Rubisco and the electron transport chain. N content for several of the measured leaves in 2008–09 dropped to values as low as 0.075 mg cm^−2^, which was not much higher than senescent foliage (0.057 mg cm^−2^). At lower foliar levels of N (0.075–0.2 mg cm^−2^), eCO_2_ leaves utilized N more efficiently (greater PNUE) compared with aCO_2_ leaves (Fig. 2B). For leaves operating at greater PNUE, the theoretical photosynthetic N requirement is lower. In our study, this would entail a lower photosynthetic N threshold in eCO_2_ leaves. Our results could not determine this threshold, as *J*_max_ and *V*_cmax_ became similarly N-restricted below ∼0.2 mg cm^−2^, independent of CO_2_ availability.

In mature leaves, the optimum N distribution between RuBP carboxylation/regeneration and electron transport may shift as a result of environmental changes (e.g. CO_2_ concentration, nutrient availability or thermal acclimation) such that the processes are co-limiting (e.g. Makino *et al.* 1994; Medlyn 1996; Hikosaka 1997). Theory predicts that the *J*_max_ : *V*_cmax_ ratio should increase under eCO_2_ (Medlyn 1996), and simulations with pine seedlings suggest that this optimization may vary seasonally (Lewis *et al.* 1996). We found no evidence of a treatment-dependent shift through time (Fig. 4). Similar work at the Duke-FACE site with *P. taeda* also found that there was no change in the slope of *J*_max_ : *V*_cmax_ due to eCO_2_ treatments (Crous *et al.* 2008). Other observations have found that *J*_max_ : *V*_cmax_ can increase slightly (∼5 %) with eCO_2_ (Medlyn *et al.* 1999; Ainsworth and Long 2007), although this change is generally smaller than predicted by theory (Medlyn 1996).

After long-term eCO_2_ treatment at the Duke-FACE site, there was a decline in the slope of *J*_max_–N and *V*_cmax_–N in pine, indicating eCO_2_ down-regulation of overall photosynthetic capacity (Crous *et al.* 2008). In contrast, at the ORNL-FACE site, there was no treatment effect on the relationships between *J*_max_–N and *V*_cmax_–N in sweetgum. Rather, the declining slope of *J*_max_ : *V*_cmax_ through time for all treatments suggests a gradual reallocation of N as plants acclimated to reductions in soil N availability.

The decline in photosynthetic capacity and resultant reduction in eCO_2_ enhancement of NPP (Norby *et al.* 2010), coupled with the demonstrated reduction in N availability for eCO_2_ plots (Garten *et al.* 2011), is consistent with the progressive N limitation hypothesis (Comins and McMurtrie 1993; Luo *et al.* 2004). New root production was stimulated in eCO_2_ plots (Norby *et al.* 2004) likely as a result of increased internal plant nutrient demand and chemical signalling, including the buildup and translocation of newly fixed sugars. Enhanced root production and exploration at deeper soil depths increased N availability and uptake for eCO_2_ trees (Finzi *et al.* 2007; Iversen *et al.* 2011), but N uptake rates were unable to meet demand and resulted in progressive reductions in photosynthesis. Limitation by other elements could also dampen photosynthetic activity, e.g. Mg, which is the central element in chlorophyll and a key cofactor for Rubisco activation. However, base cation uptake (including Mg and Ca) increased through time in eCO_2_ trees based on wood composition (K. Kim *et al.*, unpubl. data), likely as a consequence of the enhanced root exploration. Foliar content of other essential mineral nutrients such as P and K did not decline through time in litter collected from 1998 to 2002, nor in wood tissue collected in 2009.

The measurement of gas exchange under fully rehydrated conditions was useful for resolving maximum rates of photosynthesis. Under these hydrated conditions, stomatal conductance remained lower for eCO_2_ foliage than for aCO_2_ foliage; eCO_2_ : aCO_2_ *g*_s_ ranged from 0.75 to 0.81. Similarly, under dry field conditions, whole canopy conductance (*g*_c_) based on sap flow remained lower for eCO_2_ foliage; eCO_2_ : aCO_2_ *g*_c_ was ∼0.7 and declined to ∼0.4 as stomatal aperture declined during extreme drought (Warren *et al.* 2011*b*). The relationship between *A* and *g*_s_ did not change much through time for aCO_2_ foliage, but was significantly reduced for eCO_2_ foliage (Fig. 5). This suggests that the eCO_2_ stimulation of photosynthetic WUE declined through time with increasing N limitation. Even so, WUE remained greater for eCO_2_ sweetgum trees, and the relationship between stomatal conductance and assimilation was in agreement with predictions of the optimal stomatal model (Medlyn *et al.* 2011), independent of leaf N content (De Kauwe *et al.* 2013). At the Duke-FACE site, partitioning N either to ‘photosynthetically active’ or ‘storage components’ based on the *N*–*V*_cmax_ threshold allowed for linkage of eCO_2_-stimulated WUE to marginal N-use efficiency within the context of optimality theory (Palmroth *et al.* 2013). Results from these studies allow predictive capacity of ecosystem level responses to changes in site resources such as CO_2_, water and nutrients.

While shorter-term environmental (e.g. solar radiation, T, CO_2_ concentration) regulation of photosynthesis has been studied in detail, knowledge of longer-term shifts in the photosynthetic apparatus in response to eCO_2_ has been generally limited to observations at the FACE studies and a few long-term open-top chamber studies (e.g. Erickson *et al.* 2013). Long-term dynamics of soil water or nutrient availability (Finzi *et al.* 2006; Palmroth *et al.* 2013) and growth sink demands (Paul and Pellny 2003; Fatichi *et al.* 2013) provide additional regulation of photosynthetic feedback mechanisms and capacity of C uptake in some ecosystems. These processes are not always well represented in global models that depend on scaling mechanistic photosynthetic responses to the land surface (Smith and Dukes 2013). However, it is encouraging that some optimization models do successfully predict biochemical (e.g. reduced foliar N) and mechanistic (e.g. reduced stomatal conductance) process response to eCO_2_ and shifts in resource availability (Dewar *et al.* 2009). Data from the FACE studies, including this one, are being used in a multi-model comparison to assess land surface model structure and variation in predictive capacity, which will result in future model improvement (e.g. De Kauwe *et al.* 2013; Walker *et al.* 2014).

## Conclusions

Photosynthesis is the predominant mechanism that removes CO_2_ from the atmosphere. Therefore, accurate projections of CO_2_ feedbacks to climate forcing rely on the correct mechanistic representation of plant photosynthetic C assimilation capacity. At the ORNL-FACE site, the initial stimulation of carbon assimilation by sweetgum trees exposed to eCO_2_ was lost over the 12-year study. The loss of stimulation was due to the declining foliar N content, which has been related to lower soil N availability and uptake. Sweetgum foliar N content below a threshold value (∼0.2 mg N cm^−2^ leaf area) restricted carbon assimilation due to photosynthetic biochemical limitations. This study indicates that atmospheric enrichment of CO_2_ may result in an initial ‘fertilization’ effect, directly increasing photosynthesis and productivity, but this may later be offset by the declining soil N availability, which could completely eliminate the initial positive effects of eCO_2_. Overall, we suggest caution in extrapolating shorter-term eCO_2_ responses to longer-term ecosystem processes, which are confounded by other controlling factors such as soil nutrient availability, water availability or air temperature that can change through time.

## Sources of Funding

This article is based upon work supported by the US Department of Energy, Office of Science, Office of Biological and Environmental Research, under contract DE-AC05-00OR22725.

## Contributions by the Authors

J.M.W. designed and conducted the experiment. A.M.J. performed laboratory work. B.E.M. analysed the data. All authors contributed to the writing and editing of the manuscript.

## Conflicts of Interest Statement

None declared.
